# Randomized phase II trial of hypofractionated proton versus carbon ion radiation therapy in patients with sacrococcygeal chordoma-the ISAC trial protocol

**DOI:** 10.1186/1748-717X-9-100

**Published:** 2014-04-29

**Authors:** Matthias Uhl, Lutz Edler, Alexandra D Jensen, Gregor Habl, Jan Oelmann, Falk Röder, Oliver Jäckel, Jürgen Debus, Klaus Herfarth

**Affiliations:** 1 Department of Radiation Oncology, University of Heidelberg, Im Neuenheimer Feld 400, 69120 Heidelberg, Germany; 2 Heidelberg Ion Therapy Center (HIT), Im Neuenheimer Feld 450, 69120 Heidelberg, Germany; 3 German Cancer Research Center (dkfz), Im Neuenheimer Feld 280, 69120 Heidelberg, Germany

**Keywords:** Sacral chordoma, Chordoma, Carbon ion therapy, Proton therapy, Irradiation, Randomized trial, Hypofractionation, Heavy ion therapy

## Abstract

**Background:**

Chordomas are relatively rare lesions of the bones. About 30% occur in the sacrococcygeal region. Surgical resection is still the standard treatment. Due to the size, proximity to neurovascular structures and the complex anatomy of the pelvis, a complete resection with adequate safety margin is difficult to perform. A radical resection with safety margins often leads to the loss of bladder and rectal function as well as motoric/sensoric dysfunction. The recurrence rate after surgery alone is comparatively high, such that adjuvant radiation therapy is very important for improving local control rates. Proton therapy is still the international standard in the treatment of chordomas. High-LET beams such as carbon ions theoretically offer biologic advantages in slow-growing tumors. Data of a Japanese study of patients with unresectable sacral chordoma showed comparable high control rates after hypofractionated carbon ion therapy only.

**Methods and design:**

This clinical study is a prospective randomized, monocentric phase II trial. Patients with histologically confirmed sacrococcygeal chordoma will be randomized to either proton or carbon ion radiation therapy stratified regarding the clinical target volume. Target volume delineation will be carried out based on CT and MRI data. In each arm the PTV will receive 64 GyE in 16 fractions. The primary objective of this trial is safety and feasibility of hypofractionated irradiation in patients with sacrococygeal chordoma using protons or carbon ions in raster scan technique for primary or additive treatment after R2 resection. The evaluation is therefore based on the proportion of treatments without Grade 3–5 toxicity (CTCAE, version 4.0) up to 12 months after treatment and/or discontinuation of the treatment for any reason as primary endpoint. Local-progression free survival, overall survival and quality of life will be analyzed as secondary end points.

**Discussion:**

The aim of this study is to confirm the toxicity results of the Japanese data in raster scan technique and to compare it with the toxicity analysis of proton therapy given in the same fractionation. Using this data, a further randomized phase III trial is planned, comparing hypofractionated proton and carbon ion irradiation.

**Trial registration:**

ClinicalTrials.gov Identifier: NCT01811394.

## Introduction

The advantage of radiation therapy with particles is based on the particular physical and biological characteristics of ions. Protons and carbon ions as compared to photons lead to an improved conformal dose distribution, which allows a better sparing of surrounding tissue and concurrent dose escalation in the tumor. Carbon ions seem to have an additional biological advantage due to their higher biological effectiveness in contrast to protons [1-3]. Complex double-strand breaks of the DNA seem to be the reason [4,5]. Therefore, heavy ions provide a promising treatment option for tumors with lower radiosensitivity and critical location. Increased cure rates and low toxicity of a particle therapy are the expected results in tumors with low alpha/beta values. Chordomas, as a typical example, are rare malignant tumors (1-4% of all malignant bone tumors). This slow growing tumor arises from embryonic remnants of the chorda dorsalis. The incidence rate is 0.1/1,000,000 [6]. Historically, a higher incidence of sacral chordomas than skull base chordomas was assumed [7]. However, published SEER data of 400 patients with chordoma show an equal distribution with 32% on the skull base, 32.8% spinal and 29.2% in the sacral region [6]. The histological classification is divided into three types: conventional (most common type), chondroid and dedifferentiated [7-9]. The quality of surgical margin is the most important factor for local control and survival [10-12]. An adequate safety margin can only be achieved in about 50% of the patients with sacrococcygeal chordomas [11-13]. Therefore, after R1/R2 resection the local recurrence rate is about 100% without any additional therapy. Fuchs et al. could show a significantly different time of local control between patients with radical resection and subtotal resection [11]. Nevertheless the data of Park et al. indicate a large difference in local failure rate between patients irradiated for primary versus recurrent sacral chordomas after surgery. Hence, an adjuvant high-dose radiotherapy for acceptable control rates is required after complete resection [14]. High conformal techniques help to achieve a safe dose escalation. As mentioned above, due to their physical properties ions provide the best conditions. Proton therapy is the current international standard in irradiation of skull base chordomas. The results of the Loma Linda University Medical Centre, the Massachusetts General Hospital in Boston, and the PSI in Villingen (Switzerland) show excellent control rates for proton therapy [15-17]. In the GSI in Darmstadt, we irradiated with carbon ions by now about 300 patients with skull base chordoma/chondrosarcoma. Published data of the first 96 patients that were treated at the GSI displayed 3- and 5-year local tumor control rates (LC) of 81% and 70% with overall survival (OS) rates of 92% and 89%, respectively [18]. Currently, there is a randomized phase III trial under recruitment, testing protons against heavy ions in patients with skull base chordoma in our department [19]. In addition, over 70 patients with sacral chordoma were treated with carbon ion in our heavy ion therapy center (HIT) between 2010 and 2012. Furthermore, in chordomas of the sacral region surgical resection is still the standard therapy and the extent of surgical resection is important for the local disease free interval [11]. Due to the size, proximity to neurovascular structures and the complex anatomy of the pelvis a complete resection with adequate safety margin is often difficult to realize. A radical resection, especially above S2, often leads to urinary and bowel incontinence as well as motoric/sensoric dysfunctions [20,21]. An adjuvant/additive treatment with charged particles can increase the local control rate after surgery [14,22,23]. Radiobiological considerations lead to low alpha/beta value for chordoma (the tissue-specific constant that indicates the sensitivity of the tissue for the probability of late toxicity by increasing the single dose). The alpha/beta value for chordoma cells (2 Gy) [24] is significantly lower for other organs at risk (bladder alpha/beta 4.0 Gy; rectum alpha/beta 3.9 Gy). Thereby, a higher single dose can be used, treating the chordoma cells more effectively without increasing the risk of side effects. A Japanese trial with primary carbon ions irradiation without previous surgery confirmed this radiobiological model [25]. The data show an excellent control rate after primary radiotherapy with carbon ions in patients with inoperable sacral chordoma. 95 patients were treated with 16 fractions carbon ions over 4 weeks with a total dose of 52.8 GyE up to 73.6 GyE (70.4 Gy median TD). The fraction dose therefore was 3.3 GyE up to 4.6 GyE (4.4 Gy median SD) applied four times a week. At 5 years, the OS rate and LC rate were 86% and 88%, respectively. The rate of acute/late skin toxicity grade 3 and higher was 6.3%. Only one patient (1%) showed transient rectal bleeding 20 months after therapy (Grade II). The published study with ion irradiation of sacral chordoma was conducted using a passive beam modulation. A further development of the ion irradiation is the active beam delivery using raster-scanning method. The advantage of this method is especially the lower neutron production when used in our department. As a result of the higher neutron production the passive beam modulation shows no advantage over IMRT regarding secondary cancer risk [26]. Until now there have been no published data of hypofractionated irradiation with protons in sacral chordoma. Therefore, this is the first randomized Phase II trial protocol with hypofractionated carbon ion versus proton treatment in patients with sacrococcygeal chordoma in a single institution open for recruitment since January 2013.

## Methods and design

### Primary objectives/endpoints of this study

The primary objective of this trial is the evaluation of safety and feasibility of hypofractionated irradiation in patients with sacrococygeal chordoma using ions (protons or carbon ions) in raster scan technique for primary or additive treatment after R2 resection. The evaluation is based on as primary endpoint defined as the proportion of patients treated without Grade 3–5 toxicity (NCI-CTC-AE) up to 12 months after treatment and/or without discontinuation of the treatment for any reason.

### Secondary objectives

Assessment of local progression free survival (LPFS) is determined from start of treatment until local progression in imaging (>10% size increase). Further objectives are overall survival (OS) from start of treatment until death or censoring and Quality of life (QoL) using the EORTC-QLQ30 questionnaire.

### Study design

**Figure 1:**
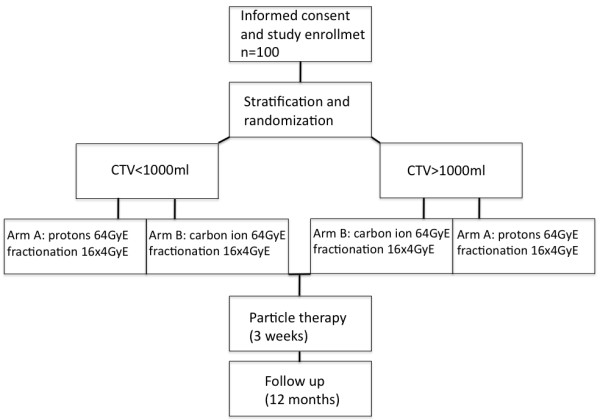
Workflow.

The study is a parallel group prospective clinical phase II trial of patients with sacrococcygeal chordoma, randomized to one of the two treatment arms (arm A: proton therapy, arm B: carbon ion therapy). A total dose of 64 GyE in 16 fractions to the PTV (see target definition below) will be given in arm A using protons and in arm B using carbon ions. The accrual period of this trial will take approximately two years with a follow up time of 12 months for each patient. Patients matching the eligibility criteria and willing to participate with informed consent are registered (Figure 1).

### Inclusion criteria

Histological confirmation of sacrococcygeal chordoma.

Karnofsky performance status ≥ 70%.

Patients age 18–80 years.

Macroscopic tumor (MRI).

Written informed consent.

### Exclusion criteria

Distant metastasis (M+).

Lack of macroscopic tumor.

Tumor extension in craniocaudal direction >16 cm due to technical limitation.

Metal implants at the level of the tumor that could influence the treatment planning.

Inability of the patient to lie quiet for at least 20 minutes (e.g. due to pain).

Prior radiotherapy of the pelvic region.

Simultaneous participation in another trial that could influence the results of the study.

Active medical implants without treatment approval at the time of ion irradiation (e.g., cardiac pacemaker, defibrillator).

### Randomization

Randomization will be performed regarding treatment arms A (protons) and B (carbon ions) stratified by the volume of CTV (>or ≤ 1 L).

### Treatment planning

Examinations for treatment planning consist of a CT scan (3 mm slice thickness) in treatment position and a MRI for 3D image correlation. The delineation of the chordoma requires a T1 weighted post gadolinium and T2 stir MRI.

### Target volume

Gross Tumor Volume (GTV) includes the gross tumor based on CT and MRI imaging. The Clinical Target Volume (CTV) is defined as GTV plus surrounding areas at risk for containing microscopic disease. The CTV includes the GTV and, after partial resection, the tumour bed and surgical access path with a margin of 2 cm in bone and soft tissue. The CTV margins may be smaller if the GTV is adjacent to the critical normal organs like small bowel or rectum. The Planning Target Volume (PTV) includes the CTV with an additional margin of 5 mm in anterior-posterior direction and 7 mm in lateral direction to compensate set-up variability. The overlap of PTV and rectum is defined as PTV-Rectum.

### Proton/carbon ion therapy

Treatment planning is realized using a treatment planning system (TPS Siemens) that enables conventional and biological optimization. After inverse planning proton and carbon ion treatment is given in active beam application (raster scanning method).

### Dose prescription

95% of the PTV should obtain the dose of 64 GyE in 16 fractions (5–6 fractions per week). The equivalent photon dose in 2 Gy/fraction (ED2) is 96 Gy, calculated for 2 Gy single dose (SD) and α/β value of 2.

### Critical normal structure constraints

**Figure 2:**
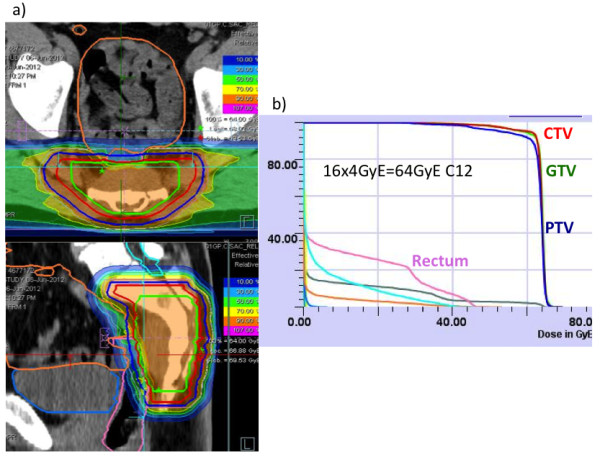
**Dose distribution and DVH of a patient treated with 64GyE carbon ion therapy within the ISAC trial. a)** dose distribution and **b)** dose volume histogram (DVH) of a patient within the trial.

Rectum, bladder and bowel are defined as organs at risk. Rectum is defined therefore ranging from the anus to the recto sigmoid junction, the bowel as the remaining intestine in the pelvis without rectum. The maximum dose to the PTV-rectum is 57.6 GyE (90% isodose). The ED2 (2 Gy SD, α/β = 3.9 Gy) is 73.2 Gy. One third of the circumference should be below the 35% isodose (ED2: 20 Gy). The maximum dose for the other parts of the intestine is 51.2 GyE (80% isodose, ED2: 61.4 Gy). This dose may only appear in a small volume. The QUANTEC criteria for the rectum and the other parts of the intestine have to be observed (V50 < 50%, V60 < 35%, V65 < 25%, V70 < 20% and V75 < 15%) (Figure 2). The maximum dose to the cauda equina is 60 Gy (ED2).

### Assessment of efficacy parameters

#### Toxicity and safety

To evaluate the toxicity, this study will use the International Common Terminology Criteria for Adverse Events (CTCAE) version 4.0 for toxicity and adverse event reporting. Safety and toxicity of the study treatment will be evaluated by clinical examination as well as imaging studies (MRI or CT). At enrollment, each patient will be asked for his medical history and pass through a basic clinical examination with regard to already existing problems. During radiation therapy the patient is monitored continuously. Once a week, a meeting with the investigator will take place in order to record the adverse effects. At the end of the treatment and in the follow up visits the clinical symptoms and toxicity will be documented again. Follow up visits are planned for 6–8 weeks, 4 months, 8 months and 12 months after irradiation. A safe feasibility exists, if no grade 3 or higher toxicity has occurred (including toxicity-related death, Grade 5) from start of radiation until 12 months after the end of therapy, and if the therapy was not canceled due to any toxicity (grade 1–4). It will be evaluated be the respective portion of patients denoted SFR (safe feasibility rate).

### Quality of life

The quality of life (QoL) is detected using the EORTC QLQ-C30 questionnaires that are filled in by the patient before treatment, at the end of treatment and 12 months after irradiation. The evaluation of the questionnaires is carried out for the individual patients after the complete collection of all data. To determine a change in QoL it will be compared with the pretherapeutic QoL for each patient in both arms. QoL will be compared between the two arms of the study after end of radiation and at end of study for the patients. For the analysis of the EORTC questionnaires the EORTC QLQ-C30 Scoring Manual will be used.

### Local progression free survival (LPFS)

The effectiveness of treatment is examined through MRI follow-ups. A progressive disease (PD) is achieved if the tumor size increases 10% or more, measured as the longest dimension in cranial-caudal, anterior-posterior and lateral direction. This definition of PD is based on the observation of a temporary tumor swelling after the particle therapy. A new tumor nodule and a tumor growth >10% in one direction (for example toward the gluteal muscles) will also be evaluated as PD. Since chordomas usually have a very low proliferation rate (Ki 67: <5%), an assessment by RECIST criteria is too insensitive for detecting a PD.

### Overall survival (OS)

**Figure 3:**
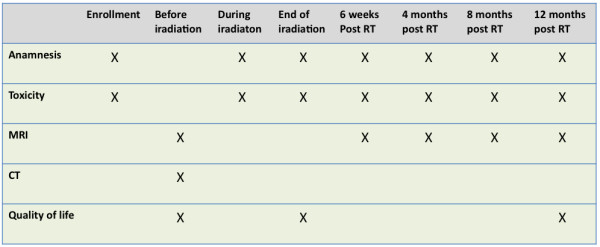
**Examinations during the trial in tabular view.** RT = radiothrapy.

Overall survival is a secondary endpoint of the study. The duration of survival is the time interval between beginning of radiotherapy and the date of death due to any cause or to loss to follow-up (censored observation). Patients not reported dead or lost to follow-up will be censored at the date of the last follow-up examination (Figure 3).

### Statistical consideration and analysis

With the present study, basic data are obtained for hypofractionated carbon ion radiation and proton radiation in sacral chordoma patients to be used for planning a confirmative randomized phase III trial comparing proton and carbon ion irradiation. At present, hypofractionated carbon ion radiation is considered as the standard, supported by data already existing in Japan. However, the treatment in Japan was performed without using the raster-scanning method. Nevertheless, these data can serve as historical control for the planning of this pilot study and provide the framework for the assessment of safety and feasibility of the proposed study treatments using the SFR as endpoint. Each of the planned study treatments (Arm A: protons or arm B: carbon ions) will be tested separately for this endpoint in a non-inferiority study design in comparison with that (historical) control. The comparative evaluation of favorable toxicity is assessed in an exploratory comparison by comparing the SFR of both arms. With these results, a further trial is then planned to evaluate the efficacy and safety of hypofractionated irradiation with carbon ions or protons with active beam delivery of sacral chordoma. Consequently, the following two questions will be considered in preparation for the subsequent confirmatory study:

a) Is the toxicity of carbon ion irradiation (Arm B) non-inferior to the current standard?

b) Is the toxicity of the proton irradiation (arm A) non-inferior to the current standard?

The number of patients to be recruited is calculated separately for each arm with an assumed standard toxicity rate of 7% (Grade 3–5 NCI-CTC AE Tox, including premature termination). Thus, the safety rate is set to 93% accounting for the Japanese results, where a toxicity of grade 3 or higher in 6.3% of patients was found [25]. Identical non-inferiority limits for toxicity and statistical error probabilities (type I error = type II error = 10%) are selected for each of the two arms. Randomization is used to obtain a balanced patient population in both groups and based on permuted block randomization stratified for the CTV size (> or ≤ 1 L). Comparison between the two arms will be carried out as a secondary comparison. The non-inferiority of each of the two arms compared to the standard is assessed in a one-sided hypothesis test: H0: SDR <93% versus H1: SDR > = 93% - DELTA. For the proposed pilot trial a non-inferiority limit (DELTA) of 13% is assumed. To test the efficacy and safety of the treatment, 45 evaluable patients per arm are required to reach a power of 90% (type II error = 10%) at a significance level 10% (type I error). Non-inferiority is denied, if safe feasibility is possible in less than 40 patients (i.e. if more than 5 of the 45 (90%) evaluable patients show toxicity. Each of the two tests is to be tested with a power of 90% (power = 90%, beta = 0.1) in a one-sample binomial test. Confidence intervals of the SFR are calculated as describing parameters. With an estimated drop out rate of approximately 10%, 50 patients should be included in each arm in order to obtain 45 evaluable patients. This sample size calculation is based on the PASS software of 2005 and the method of Blackwelder (1982) for non-inferiority trials [27]. The secondary endpoints OS and LPFS will be analyzed via descriptive methods of censored survival times (Kaplan Meier method).

### Regular study end

The estimated accrual period is 24 months. The regular end of the treatment period for each patient is three weeks after initiation of radiation therapy (after 16 fractions). The regular end of study participation for each patient is after a follow up period of 12 months.

### Prematurely study termination

Reasons for premature termination of the entire study are unacceptable risks and toxicities as specified by the Safety Board as occurrence of a toxicity of grade 5, of 2 consecutive grade 4 toxicities or 5 consecutive grade 3 toxicities judged to be definitively associated with study therapy. Other reasons are new scientific findings during the period of study that require a different treatment. Individual reasons for premature study termination are serious events or a patient’s request.

### Data safety monitoring board (DSMB)

An independent Data and Safety Monitoring Board (DSMB) will monitor the recruitment, the reported adverse events and the data quality. Based on its review the DSMB will provide the Principal Investigator (PI) with recommendations regarding trial modification, continuation or termination.

### Data collection and management

All patient related data are collected pseudonymously. An individual patient number characterizes each patient. The data collection is based on case report forms (documentation forms/case report forms). The originals of all documents are kept in the study center. According to the §13 of the German GCP-Regulation, all important trial documents will be archived for at least 15 years after the end of the ISAC trial. According to the §28c of the German X-Ray Regulation (RöV) and the §87 of the German Radiation Protection Regulation (StrlSchV) the informed consent forms including the patients’ consent for trial participation and the application of irradiation will be archived for at least 30 years after the end of the trial. The Study Center at the Department of Radiation Oncology will be responsible for archiving all relevant data.

### Declaration of Helsinki and good clinical practice

The trial is conducted in accordance with the Declaration of Helsinki (2008 Version of the Declaration of Helsinki, adopted at the 59th WMA General Assembly, Seoul, October 2008) as well as with the guidelines of Good Clinical Practice (s. ICH-GCP: International Conference on Harmonization - Good Clinical Practice; 01.05.1996) in their current versions.

### Ethics Committee and Bundesamt für Strahlenschutz

The study protocol, patient information and consent are approved by the ethics committee of the university of Heidelberg (S-165/2012). Furthermore, the trial is approved by the Bundesamt für Strahlenschutz (Z5-22461/2-2009-026/13).

## Discussion

Currently, radical resection with/without adjuvant radiation treatment is the most performed treatment in patients with sacral chordoma. Hence, the evidence based only on small retrospective series [11,14]. De Lany et al. published data of 29 patients with chordomas of the mobile spine and sacrum that were treated by surgery and high dose proton-/photon irradiation in a phase II trial. In this trial no significant difference between R0 and R1/R2/biopsy could be shown regarding local control [28]. According to these results it can be asked whether an extended surgery with high morbidity is still necessary. Japanese retrospective data compared surgery only with carbon ion therapy only and found out a 5-year local-recurrence free survival rate of 62.5% for surgery and 100% for carbon ion therapy [22]. Further retrospective data of 95 patients after hypofractionated carbon ion treatment at NIRS seems to indicate a new therapeutic option in patients with sacral chordoma, which displays in contrast to the surgery lower morbidity with very good local control [25]. Imai et al. reported about three patients with grade 3 skin toxicity and two patients with grade 4 (73.6 GyE) [25]. A decided evaluation of skin toxicity after carbon ion treatment at NIRS showed grade 3 and 4 skin toxicity only in patients which received doses ≥ 70 GyE in 16 fractions. It was indicated that severe skin reaction may not develop if the prescribed dose is 64 GyE or less like in our trial [29]. Furthermore, we limit the maximum dose of the skin to 90-95% of the prescribed dose. Imai et al. reported about one patient with rectal bleeding grade 1, but no patient received a colostomy or urinary diversion after carbon ion treatment. 15 patients (16%) required medication due to sciatic neuropathy after carbon ion treatment. However, five of them received with 73.6 GyE a much higher dose than in our trial. More than 80% of the chordomas were localized above S2 [25]. Thus, the expected toxicity after radical resection would have been a complete urinary and bowel incontinence in at least 80% of the patients [20,21]. Furthermore chronic neuropathic pain, wound complications and walking difficulties are also possible side effects of sacrectomy [30,31]. Against this background, the published side effects after hypofractionated carbon ion therapy should be evaluated.

Due to missing data for a hypofractionated carbon ion therapy in raster scan technique, this trial will confirm the historical data from Japan that were developed by using passive beam application. In the Japanese trial patients received a total dose of 52.8 to 73.6 GyE in 16 fractions. Most of the patients received 70.4 GyE. In contrast to our fractionation with up to 6 fractions per week (Monday-Saturday), the patients in Japan received only four fractions per week. Therefore, the total dose in our trial is limited to 64 GyE. Only patients with inoperable, residual or recurrent tumor will be included in our trial. Patients with resectable tumor are included only after refusal of surgery treatment. Moreover, no data of hypofractionated proton therapy in patients with sacral chordoma have been published so far. However, these data are necessary to perform a randomized prospective phase III study in the future.

## Competing interests

The authors declare that they have no competing interests.

## Authors’ contributions

MU, LE, ADJ, JD und KH have developed the study concept. MU, LE, KH and JD wrote the study protocol and obtained ethics approval. LE was responsible for statistical considerations. MU, ADJ, GH, JO, MBFR, JD and KH will provide patient care. MU, LE, ADJ, GH, JO, MBFR, OJ, JD and KH will implement the protocol and oversee collection of the data. All authors contributed to and approved the final manuscript.
